# Potential value of pre- and post-therapy [68Ga]Ga-DOTA-TATE PET/CT in the prognosis of response to PRRT in disseminated neuroendocrine tumors

**DOI:** 10.3389/fendo.2022.929391

**Published:** 2022-08-15

**Authors:** Marta Opalińska, Karolina Morawiec-Sławek, Adrian Kania-Kuc, Ibraheem Al Maraih, Anna Sowa-Staszczak, Alicja Hubalewska-Dydejczyk

**Affiliations:** ^1^ Nuclear Medicine Unit, Endocrinology Department, University Hospital in Krakow, Krakow, Poland; ^2^ Chair and Department of Endocrinology, Jagiellonian University Medical College, Krakow, Poland

**Keywords:** neuroendocrine tumors, NET, PRRT, [68Ga]Ga-DOTA-TATE PET/CT, SUVlmax, SUVlmean, outcome prediction

## Abstract

**Background:**

Peptide receptor radionuclide therapy (PRRT) is one of the most effective therapeutic options for the treatment of metastatic, well-differentiated neuroendocrine tumors (NETs). It improves progressive disease-free survival and enables the control of hormone secretion in functioning tumors.

Currently, there are no clearly established predictors of response to PRRT. The main factors hindering such a prediction are the heterogeneity of somatostatin receptor expression within and between lesions, lack of standardized parameters for functional imaging, and the use of different PRRT protocols.

The main goal of our study was to quantify SUVmax changes in [68Ga]Ga-DOTA-TATE PET/CT scans as a potential predictor of long-term response to PRRT.

**Material and methods:**

Out of 20 patients treated with PRRT using [177Lu]Lu and/or [177Lu]Lu/[90Y]Y-DOTA-TATE in 2017–2019 due to dissemination of neuroendocrine neoplasm, 12 patients underwent [68Ga]Ga-DOTA-TATE PET/CT on average 3.1 months before and 4.5 months after PRRT and were eligible for the analysis.

In total, 76 NET lesions were evaluated. We measured SUVmax for every lesion in both PET/CT scans (before and after PRRT). Those values were corrected by liver SUVmax and liver SUVmean measured in volumetric analysis and specified as SUVlmax and SUVlmean. As a next step, changes in SUVlmax and SUVlmean were assessed based on both PET/CT scans. Finally, results were correlated with the clinical outcome assessed as progressive disease, disease stabilization, or partial response.

**Results:**

The mean follow-up period was 19.9 months. Progressive disease, partial response, and disease stabilization were found in five, two, and five patients, respectively. Among patients with a partial response, the decrease in mean SUVlmax was 66.3% when compared to baseline. In patients with stable disease, the decrease in SUVlmax was 30.3% when compared to baseline. In patients with progressive disease, the mean increase in SUVlmax was 9.1% when compared to baseline. The changes in SUVlmean were -69,8%, -30.8%, and -3.7%, respectively.

**Conclusions:**

A decrease in the SUVmax value in NET lesions, corrected by normal liver tissue uptake assessed in [68Ga]Ga-DOTA-TATE PET/CT scans, indicates a lower risk for NET progressive disease within 20 months after PRRT and may constitute an additional and independent parameter for the estimation of overall risk for disease progression.

## Introduction

Neuroendocrine tumors (NETs) constitute a heterogeneous group of neoplasms, most of which are well-differentiated (G1 and low G2 according to the WHO classification) and characterized by relatively slow growth, which is associated with a fairly favorable prognosis. However, NETs with a higher Ki67 level are usually more aggressive and tend to grow faster, resulting in poorer outcomes and an impairment in quality of life.

Overexpression of somatostatin receptors on NET cell surfaces is a common feature of NETs, which enable a theranostic approach with the use of somatostatin analogues (SSA) in diagnosis and therapy. The basic molecular methods of NET imaging include [68Ga]Ga–DOTA-peptide (TATE/TOC/NOC) PET/CT or [99mTc]Tc-HYNIC-peptide (TOC/TATE) SPECT/CT. Recently published American and European guidelines (1, 2) recommend the use of [68Ga]Ga-DOTA-conjugated peptide PET/CT as the method of choice in imaging, staging, restaging, and the determination of somatostatin receptor (SSTR) status; however, no consensus was reached in choice of imaging modality regarding monitoring response to therapy after peptide receptor radionuclide therapy (PRRT) (3). This is due to the increased sensitivity of this method in comparison to other imaging methods.

In several clinical trials, PRRT was shown to be one of the most effective systemic therapeutic options in the treatment of disseminated NETs. It has been demonstrated to be effective in prolonging progression-free survival (PFS) (4). In the NETTER-1 prospective, randomized phase 3 trial, there was no significant overall survival (OS) benefit in patients with small intestine NETs treated with PRRT when compared to high doses of long-acting octreotide. However, according to the authors, despite the fact that final OS did not reach the level of statistical significance, the difference in median OS with PRRT versus high-dose long-acting octreotide alone might be considered clinically relevant (5). Nevertheless, the response to treatment differs substantially among patients. Tumor grading, Ki67 level, status of SSTR expression (6), and presence of FDG-avid metastases (7) have the same predictive value in the prediction of response to PRRT, but their effectiveness is not satisfactory. The multigenomic blood mRNA biomarker (NETest) and PRRT predictive quotient (PPQ) seem to have the best predictive value (8), but their availability is still limited. Tumor growth rate (TGR) has also been shown to be a valuable option for the prediction of response to treatment in NETs (9), but this has not yet been validated for PRRT. Heterogeneity of somatostatin receptor expression within and between lesions may be one of the reasons for a diverse response to PRRT (10, 11). Molecular imaging, which is based on the evaluation of SSTR expression, can provide an accurate estimation of PRRT efficacy.

According to the literature, PET/CT parameters based on corrected values of SUVmax such as the tumor-to-blood ratio or tumor-to-spleen and tumor-to-liver ratios are suggested to be more reliable than the absolute SUVmax value, which reflects SSTR expression only in the one pixel with the greatest uptake (12) and does not take into account the variability resulting from patient weight, radiopharmaceutical dose, and total disease burden. For similar reasons, the use of volumetric parameters of SSA uptake should provide more reliable information about the mean SSTR expression in the tumor tissue (13, 14) and may reflect its heterogeneity more precisely, which translates into an overall response to treatment (15).

The main aims of our study were to evaluate changes in corrected SUVmax in NET lesions in response to PRRT and to asses if those changes could be a predictor of response to PRRT.

## Material and methods

### Study design

Our study is a retrospective analysis evaluating the change in corrected SUVmax in NET lesions assessed by [68Ga]Ga-DOTA-TATE PET/CT performed before and after PRRT.

### Study population

Patients treated with PRRT in the Nuclear Medicine Unit, Endocrinology Department, University Hospital in Krakow, between 2017 and 2019 were retrospectively analyzed.

The inclusion criteria for the current analysis were as follows: diagnosis of NET confirmed by histopathology (advanced stage of disease not amenable to curative resection), qualified for PRRT due to progressive disease, available imaging data from [68Ga]Ga-DOTA-TATE PET/CT before and after PRRT, presence of at least one target lesion.

The exclusion criterion for the current analysis was presence of concomitant malignancy.

Clinical data were collected from an electronic database and is presented in **Table 1**.

**Table 1:** The clinicopathologic characteristics of the study group.

No.	Gender	Age(years)	Primary tumor location (NET)	Grading according to WHO	Location of metastases	Treatment prior to PRRT	PRRT type, summary dose MBq	PFS (months)	OS(months)
1.	F	73	Pancreas	G2	Lymph nodes, liver, and bones	Resection of the tail with partial body excision of pancreas, ablation of meta lesions in the liver, Somatostatin analogue	[177Lu]Lu-DOTA-TATE 11100 MBq	54	Alive
2.	M	68	Lung	G2	Right lung, lymph nodes, left ventricular muscle	Somatostatin analogue	[177Lu]Lu-DOTA-TATE 12950 MBq [90Y]Y/[177Lu]Lu-DOTA-TATE 7400 MBq	19	36
3.	F	44	Rectum	G1	Lymph nodes and bones	Anterior rectal resection, Somatostatin analogue	[90Y]Y/[177Lu]Lu-DOTA-TATE 11100 MBq	29	Alive
4.	F	63	Rectum and anal canal	G2	Bones, lungs, and liver	Somatostatin analogue	[90Y]Y/[177Lu]Lu-DOTA-TATE 9250MBq [177Lu]Lu-DOTA-TATE 3700 MBq	42	Alive
5.	F	77	Small intestine	G2	Peritoneum (at the anterior abdominal wall and pelvis and around the liver)	Chemotherapy (carboplatin + etoposide) Somatostatin analogue	[177Lu]Lu-DOTA-TATE 12950 MBq	42	Alive
6.	F	83	Pancreas	n/a	Liver	Somatostatin Analogue	[177Lu]Lu-DOTA-TATE 9250 MBq	34	Alive
7.	F	53	Pancreas	G3	Liver and lymph nodes	Chemotherapy (cisplatin + etoposide) Somatostatin analogue	[90Y]Y/[177Lu]Lu-DOTA-TATE 11100 MBq	18	Alive
8.	F	51	Pancreas	G2	Liver, lymph nodes, and the body of the Th8 vertebrae	Somatostatin analogue	[90Y]Y/[177Lu]Lu-DOTA-TATE 11100 MBq	9	Alive
9.	M	52	Small intestine	G1	Liver and lymph nodes	Partial resection of the small intestine, Somatostatin analogue	[90Y]Y/[177Lu]Lu-DOTA-TATE 14800 MBq	20	Alive
10.	M	37	Small intestine	G2	Liver and lymph nodes	Partial resection of the small intestine Somatostatin analogue	[90Y]Y/[177Lu]Lu-DOTA-TATE 14800 MBq	12	Alive
11.	M	74	Pancreas	G1	Liver	Somatostatin analogue	[90Y]Y/[177Lu]Lu-DOTA-TATE 11100 MBq	29	Alive
12	K	77	Medullary thyroid cancer	Ki67 21.4%	Liver	Complete thyroid removal with lymphadenectomy of the central neck and modified lateral lymphadenectomy on the left side Somatostatin analogue	[177Lu]Lu-DOTA-TATE 11100 MBq	10	Alive

The acquisition data for each patient were collected retrospectively from the results of [68Ga]Ga-DOTA-TATE PET/CT examinations. Prior to inclusion into our analysis, all NET lesions were evaluated for SSTR-positivity on baseline SSTR imaging (to exclude the effect of SSTR-negative lesions on PRRT results).

### Methods

#### Protocol of [68Ga]Ga-DOTA-TATE PET/CT examinations

All analyzed scans were performed using a standard protocol for [68Ga]Ga-DOTA-TATE PET/CT on a GE Discovery 690 VCT scanner. All patients were given a one-time laxative and were on a liquid diet for 1 day prior to the examination. The imaging study was performed while fasting. According to local practice, long-acting SSAs were withdrawn 3–4 weeks prior to PET/CT to avoid possible SSTR blockade. Imaging was performed 60 min after intravenous administration of the [68Ga]Ga-DOTA-TATE (150 MBq). Initially, a localizing scan was performed for planning the exam, while standard imaging extended from the mid-thigh to the top of the head. A low-dose CT without contrast enhancement (Smart mA with a range of 80–180 mA and a noise index of 17.0) was performed for photon-attenuation correction and co-localization of radiotracer uptake and anatomical structures. PET scans were performed with a 3.3-mm short-axis slice set, acquired at 3 min per bed position in three-dimensional mode. Data from PET scans were reconstructed using the GE reconstruction algorithm (matrix size 256 × 256, VUE Point FX reconstruction method: 16 subsets, 3 iterations). All images were transferred to Advantage Windows Workstation (AW 4.6, GE Healthcare), and VOI and SUV values were counted using Advantage Windows Volume Viewer Software (v.11.3, GE Healthcare).

The follow-up [68Ga]Ga-DOTA-TATE PET/CT examinations were performed on average 3–6 months after the completion of PRRT and later every 3–6 months. The shortest time of follow-up in the whole group was 19.9 months, and therefore, the analysis was performed at this point in time. During follow-up, all patients were treated with a long-acting SSA.

#### Method of corrected SUVmax assessment and analysis

**Figure 1:**
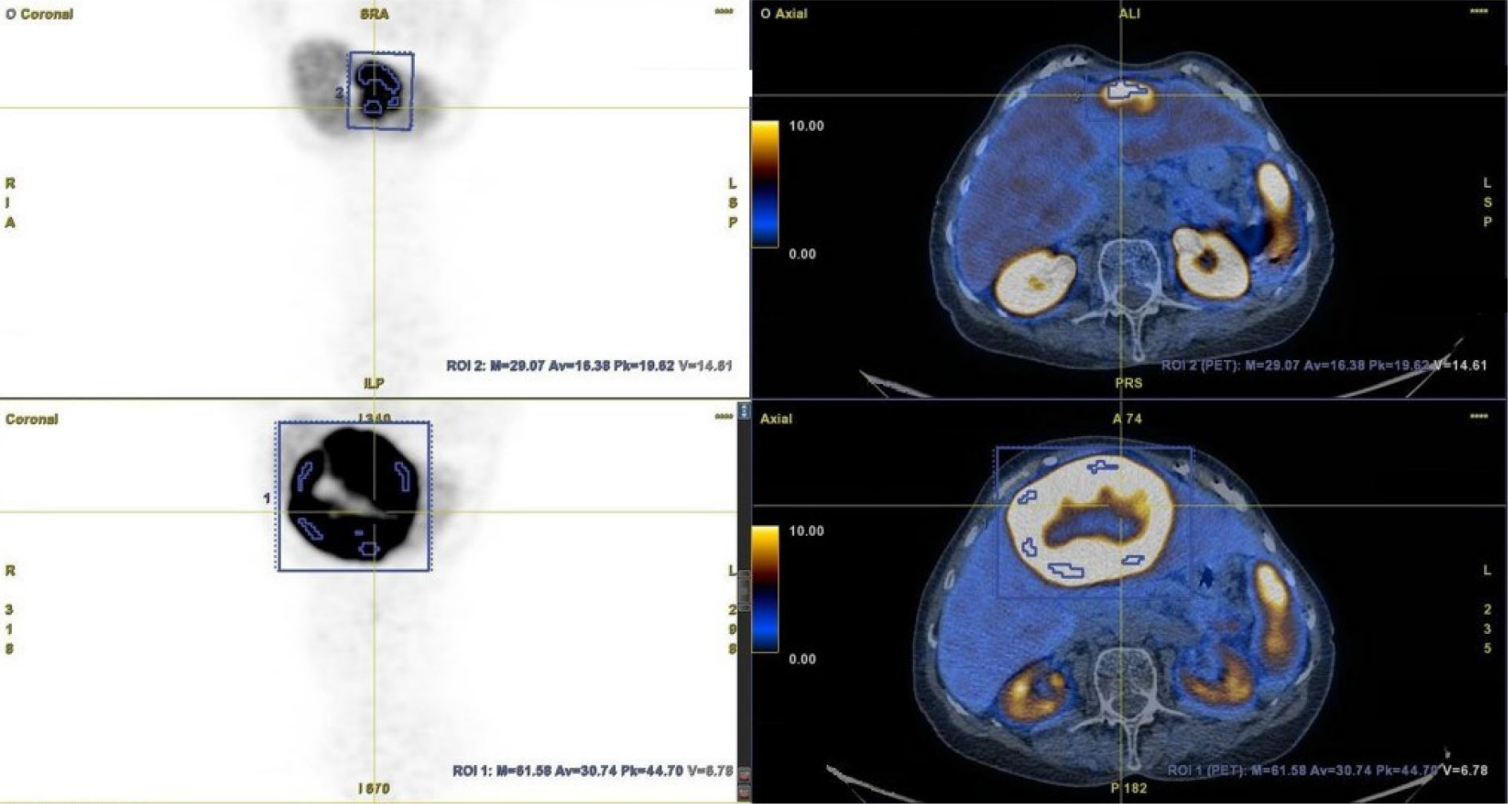
Method of metastatic lesion segmentation. [68Ga]Ga-DOTA-TATE PET/CT before PRRT (bottom row) and after PRRT (upper row).

All results from [68Ga]Ga-DOTA-TATE PET/CT scans performed before and after PRRT were assessed by two independent nuclear medicine specialists. NET lesions with increased SSA uptake [Krenning score 2, 3, or 4 (16)] and a clear margin were suitable for analysis. For each NET lesion, SUVmax was obtained in both PET/CTs. Additionally, SUVmax and SUVmean of the reference liver tissue were also obtained (**Figure 1**).

To obtain the SUVmax of NET lesions in each case, tumors were contoured with margins (if possible), taking care not to include other adjacent lesions. The SUVmean of the reference liver tissue was derived from the volume of interest (VOI), which was adjusted for the patient. If possible, the VOI was obtained from the center of the right lobe of the liver, with a specified distance from the liver edge (to avoid respiratory artefacts). In cases of massive liver involvement by metastases, the VOI was obtained from a different (usually smaller) area of the healthy liver. The SUVmax of each NET lesion in both PET/CT studies was corrected (as a quotient) to the SUVmax of the normal liver (SUVlmax) and to the mean SUV of the normal liver (SUVlmean). Furthermore, intra-patient changes in SUVlmax and SUVlmean were calculated for each NET lesion and reported as percentages. These values were also calculated altogether for NET lesions categorized by organ location (liver, pancreas, chest (lungs and heart), bone, and lymph nodes (abdominal, pelvic, and chest)) and as a mean value for each patient. Finally, this percentage change in corrected SUVs was evaluated in subgroups depending on the response to PRRT and assessed as partial response (PR), disease stabilization (SD), or progressive disease (PD) in a follow-up examination performed at least 19.9 months after PRRT. As a standard of care, the first follow-up examination was performed on average 3–6 months after the completion of PRRT (according to recommendation of the Polish Network of Neuroendocrine Tumours) and then every 3–6 months (17). Response to PRRT was assessed by contrast-enhanced CT or PET/CT with [68Ga]Ga-DOTA-TATE. Progressive disease was diagnosed if new foci of disease were found in any of the radiological studies or there was a progression seen in contrast-enhanced CT. Disease stabilization was diagnosed when the total disease burden was similar as assessed by contrast-enhanced CT or [68Ga]Ga-DOTA-TATE PET/CT. Partial response was diagnosed when regression was seen in contrast-enhanced CT.

#### Statistical analysis

The statistical analysis was performed using PSPP 1.5.3. Descriptive statistics of demographic and clinical characteristics are provided as median, range, and percentage values. The values of [68Ga]Ga-DOTA-TATE PET/CT parameters (SUVmax, SUVlmax, and SUVlmean) are provided as mean values and ranges. Differences between groups of values of SUVlmax and SUVlmean before and after treatment were calculated using paired t-tests and the Wilcoxon test as indicated. Results were considered to be statistically significant when p < 0.05.

## Results

Twelve of the 20 disseminated NET patients treated with PRRT using [177Lu]Lu and [177Lu]Lu/[90Y]Y-DOTA-TATE between 2017 and 2019 were eligible for analysis. In this group, [68Ga]Ga-DOTA-TATE PET/CT was performed 3.1 [mean, with a range of 0.4–8.8] months before and 4.5 [mean, with a range of 2.1–7.5] months after PRRT. At the time of statistical analysis, the shortest follow-up period was 19.9 months, and therefore at this point the efficacy of PRRT was assessed in all patients. During this period, progressive disease was found in five patients, disease stabilization in five patients, and partial response in two patients. From this group of 12 patients, 76 NET lesions located in the liver, pancreas, lung and heart, bone, and lymph nodes (abdominal, pelvic, and chest) were suitable for analysis. The mean SUVmax of the liver was 5.8 [3.3–9.2] and SUVmean was 4.2 [2.6–7.2]. The mean volume of the analyzed liver tissue was 9.6 cm^3^ [2.6cm^3^–56.1cm^3^]. The initial mean SUVmax for all analyzed lesions was 28.3 and decreased after PRRT to 8.3. The same analysis showed that the initial values of SUVlmax and SUVlmean were 5.9 and 8.7, whereas follow-up values were 3.2 and 4.3. Analysis based on clinical outcome showed a decrease in mean values of uncorrected SUVmax, SUVlmax, and SUVlmean in PR and SD subgroups; however, the degree of its decline depended on responses to PRRT. The mean values of uncorrected SUVmax as well as intra-patient SUVlmax in the PD group were increased when compared to baseline (**Tables 2**, **3**).

**Table 2:** Mean values of SUVmax, SUVlmax, and SUVlmean counted on [68Ga] Ga-DOTA-TATE PET/CT for individual patients in all NET lesions before PRRT and after PRRT in relation to the response to PRRT.

Response to PRRT	Mean SUV max before PRRT	Mean SUV lmax before PRRT	Mean SUVl mean before PRRT	Mean SUV max after PRRT	Mean SUVl max after PRRT	Mean SUVl mean after PRRT	Number of patients/Number of lesions
Partial response	38.2 (5.0-124.3)	7.0 (1.4-16.3)	11.2 (2.0-26.8)	12.9 (1.6-57.2)	1.8 (0.3-8.4)	2.6 (0.3-12.0)	2/18
Disease stabilization	23.6 (5.8-85.0)	5.4 (1.3-25.5)	6.5 (1.6-31.9)	18.0 (2.1-42.0)	3.4 (0.6-11.5)	4.1 (0.6-11.5)	5/31
Progressive disease	21.7 (5.7-61.2)	5.4 (0.9-17.5)	8.6 (1.4-25.7)	24.4 (6.0-36.0)	4.8 (1.2-7.6)	7.2 (1.6-12.4)	5/27
Mean	28.3	5.9	8.7	17.7	3.2	4.3	-

**Table 3:** Mean change of SUVlmax and SUVlmean counted on [68Ga]Ga-DOTA-TATE PET/CT performed before and after PRRT in relation to PRRT.

Response to PRRT	Change of SUVlmax	Change of SUVlmean	Number of patients
Partial response	-66.3% (p = 0.453)	-69.8% (p = 0.271)	2
Disease stabilization	-30.32% (p < 0.01)	-30.8% (p < 0.01)	5
Progressive disease	9.1% (p < 0.01)	-3.7% (p < 0.01)	5

**Figure 2:**
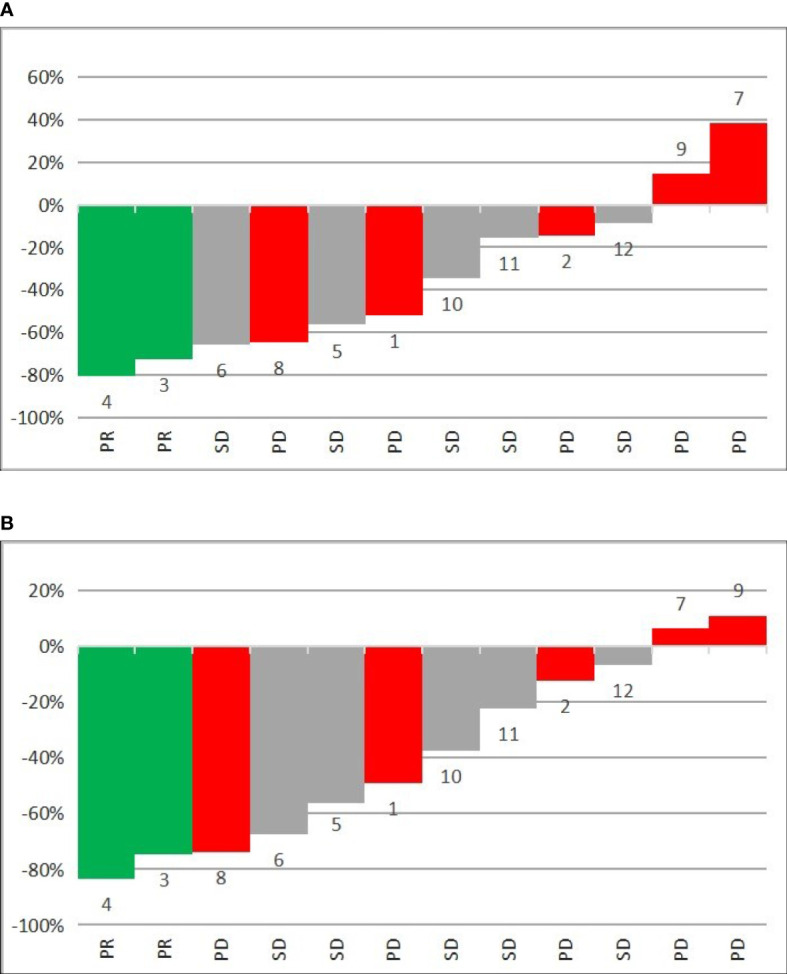
Waterfall plots of mean percentage change in 68GA-DOTA-TATE uptake in all metastatic lesions per patient of **(A)** SUVlmax and **(B)** SUVlmean before and after PRRT. PR—partial response, SD—disease stabilization, PD—progressive disease, 1–12—number of patient.

The analysis of all lesions showed an intra-patient change in SUVlmax and SUVlmean values after PRRT. The mean percentage change in SUVlmax lesions was –34.4% [-94.2%–92.3%], and that for SUVlmean lesions was -38.9% [-95.0%–47.6%] (**Table 3**). The results of SUVlmax and SUVlmean changes divided according to the response to PRRT in each patient are shown in **Figures 2A, B**. Analysis of the entire group revealed 56 lesions with a decrease in SUVlmax and 20 lesions with an increase in SUVlmax (-53.2% [range -94.2% to – 4.4%] and 20.6% [range 0.4% to 92.3%], respectively). The same analysis showed a decrease in SUVlmean in 61 lesions (mean value -51.4% [range -95.0% to -5.7%]) and an increase in SUVlmean in 15 lesions (mean value 15.2% [range 2.3%–47.6%]). The use of SUVlmean revealed 9% more lesions with a decrease in corrected SUV values when compared to SUVlmax.

Analyses of changes in SUVlmax and SUVlmean in separate organs revealed different values for different organs, with corresponding values in all analyzed groups. The greatest decrease in SUVlmax in PR patients was seen in lymph nodes, whereas the greatest decrease in mean value of SUVlmax was seen in the pancreas and bones (**Table 4**). The same pattern was observed for SUVlmean (**Table 5**).

**Table 4:** Percentage change of SUVlmax per organs affected by NET on [68Ga]Ga-DOTA-TATE PET/CT performed before and after PRRT.

The group of organs	Percentage decrease in lesions	Percentage increase in lesions	Partial response	Disease stabilization	Progressive disease	Mean	Number of lesions
Liver	-48.7%	20.3%	-56.3%	-20.4%	6.5%	-20.4%	39
Lymph nodes	-44.3%	47.0%	-90.0%	-35.2%	47.0%	-37.3%	13
Bones	-69.6%	6.2%	-73.0%	-41.9%	12.0%	-60.7%	17
Pancreas	-53.9%	–	-58.3%	-52.4%	–	-53.9%	6
Chest (lungs and heart)	-33.2%	27.0%	–	-13.2%	–	-13.2%	4

**Table 5:** Percentage change of SUVlmean per organs affected by NET on [68Ga]Ga-DOTA-TATE PET/CT performed before and after PRRT.

The group of organs	Percentage decrease in lesions	Percentage increase in lesions	Partial response	Stabilization	Progression	Mean	Number of lesions
Liver	-43.8%	15.9%	-61.0%	-21.2%	-5.5%	-27.0%	39
Lymph nodes	-46.1%	12.8%	-91.2%	-37.0%	12.8%	-41.5%	13
Bones	-70.9%	5.5%	-75.2%	-39.9%	8.4%	-62.0%	17
Pancreas	-56.4%	–	-69.0%	-52.2%	–	-56.4%	6
Chest (lungs and heart)	-31.7%	29.9%	–	-11.2%	–	-11.2%	4

## Discussion

The results of the study revealed that a change in corrected SUVmax values (SUVlmax, SUVlmean) in neuroendocrine lesions assessed before and after PRRT in [68Ga]Ga-DOTA-TATE PET/CT may indicate a lower risk for NET progressive disease within 20 months after PRRT.

Although several prognostic factors of response to PRRT in the treatment of NETs are known (18), identification of predictive factors is more challenging. One likely reason for a diverse response to PRRT is the heterogeneity of SSTR expression within and between NET lesions, resulting in various tumor phenotypes (19). This leads to differences in tumor radiosensitivity and severity of DNA damage during the treatment (20). To date, few studies evaluating the role of SSTR expression assessed by SSTR imaging in response to PRRT have been published (21). These studies have assessed various PET/CT indicators in different patient populations, making a direct comparison of their results difficult. Some of the studies showed that greater tracer uptake (expressed as SUVmax value) on pretreatment PET/CT scans could be a marker of response to PRRT (22–24). In this context, the usefulness of pretreatment [68Ga]Ga-DOTA-peptide PET/CT as a test to qualify patients for PRRT is high, but it has not been validated as an indicator to predict the degree of treatment response. In contrast, there are also studies assessing the SUVmax value in the prediction of response to PRRT, which reports negative results (25, 26).

In theory, a promising solution for the prediction of response to PRRT is a comparison of [68Ga]Ga-DOTA-peptide PET/CT parameters obtained before and after PRRT, which may show a change in SSTR expression induced by that treatment. However, such an assessment requires consideration of several issues. Differences in patient weight, administered radiopharmaceutical dose, and total disease burden may affect SUVmax measurement of tumor tissue. In part, this technical parameter-dependent SUVmax variability can be minimized by correcting its values by tracer uptake in healthy, reference tissue. The analysis performed in our study showed that, in some patients, the change in uncorrected SUVmax values has an opposite direction than the corrected one (mainly when liver SUVmax and SUVmean values were significantly reduced after PRRT). For this reason, uncorrected and corrected SUVmax cannot be used alternatively. Another important issue is the choice of reference tissue. Up-to-date tumor-to-blood, tumor-to-spleen, and tumor-to-liver ratios are the most commonly used (27). The selection of SUVmax or SUVmean of reference tissue to calculate these parameters may partially affect their values; however, the values obtained using SUVmean should be more reproducible. Matching the VOI value in cases of extreme liver involvement by metastatic lesions may, in theory, present some technical difficulties. In our analysis, the smallest volume of reference liver tissue was 2.6 cm^3^, which appears to be sufficient for reliable SUVlmean calculation. The use of spleen tissue, in which NET metastases are very rare, could solve the problem of standardizing the VOI of the reference tissue.

To achieve the aims of the study, we performed a two-step analysis. The first step showed that changes in SUVlmax and SUVlmean were different in metastases located in different organs (**Tables 4**, **5**); however, the degrees of changes in SUVlmax and SUVlmean in particular tissues were similar (**Table 3**). The greatest decrease in SUVlmax was seen in the chest (lungs and heart) in PR patients, whereas the greatest decrease in mean SUVlmax value for the whole study population was seen in the pancreas and bones (**Table 4**). There are no data in the literature to compare with our findings. The value of this observation is difficult to determine; however, it may be possible in the future to assess which metastatic location is associated with the best response to PRRT and to ascertain whether the location of the metastatic tumor determines the overall response to treatment.

Having demonstrated significant differences in SUVlmax and SUVlmean values before and after PRRT, we tested whether the degree of these changes depends on the overall response to treatment. To include in our analysis the potential heterogeneity and differences in response to radiation of metastases located in different tissues, we analyzed the mean SUVlmax and SUVlmean for each patient (counted together for all NET lesions). The results showed that both of proposed indicators are similarly robust as predictors of response to PRRT. We observed a greater decrease in SUVlmax and SUVlmean values in patients with better response to treatment, although the degree of SUVlmean decrease was slightly higher (**Table 3**). This result is probably caused by the less precise assessment of tracer uptake in liver tissue performed with use of liver SUVmax, which is based on measurement of only one pixel value.

A 2020 review article found only one study comparing the change in SUVmax parameters in [68Ga]Ga-DOTA-TATE PET/CT studies before and after PRRT (27). In this study, SUVmax and tumor-to-spleen SUV ratio (SUVT/S) were investigated in early response prediction after PRRT by evaluating changes between baseline and interim [68Ga]Ga-DOTA-TATE PET/CT, performed 3 months after the first cycle of PRRT (28). In this study, the decrease in SUVT/S predicted the patient outcome which is consistent with the obtained results. The change in uncorrected SUVmax was not correlated with treatment response, which is also consistent with our observations. Unfortunately, the authors did not repeat their analysis after completion of PRRT. Another study which evaluated 91 patients and multiple quantitative parameters assessed at baseline and after 1 cycle of PRRT showed that the degree of SSTR type 2 expression and tumor heterogeneity were predictive of the therapy response or PFS. However, changes in these parameters after cycle 1 of PRRT did not correlate with clinical outcomes (29). The mechanism of SUVlmax, SUVlmean, and SUTV/S decrease after PRRT cannot be explained by a simple loss of SSTR expression, which is related to NET differentiation. This is because their decrease correlated with a better treatment response. This could be partially explained by the change in velocity of radiotracer clearance from reference organs, but this hypothesis requires further study and comparison with radiotracer clearance from pool blood.

To test the comparability of using spleen as a reference tissue, we counted the corrected SUVmax values using the SUVmax and SUVmean of spleen as a reference tissue [data not shown]. The results showed similar results as SUVlmax and SUVlmean but were statistically not significant, probably because of the too small group size.

In summary, our study revealed that changes in SUVlmax or SUVlmean may have clinical relevance for identifying NET patients at a higher risk of progression after PRRT. Both proposed parameters are easy to obtain, are highly reproducible, and are not susceptible to inter-reader variability. The only limitation is the need to perform both PET/CT examinations on the same scanner and to use the same imaging protocol.

Because the investigation is a small pilot study with only 12 patients with different primary NET locations included, the results should be confirmed in a larger cohort, preferably in a prospective setting.

## Conclusions

A greater decrease in total (counted for all NET lesions) values of corrected SUVmax (SUVlmax, SUVlmean) assessed by [68Ga]Ga-DOTA-TATE PET/CT performed before and after PRRT may indicate a lower risk for NET progressive disease at 20 months after PRRT. It might also constitute an additional independent parameter in the estimation of risk for progressive disease in this group of patients.

## Data availability statement

The original contributions presented in the study are included in the article/supplementary material. Further inquiries can be directed to the corresponding author.

## Ethics statement

The study protocol was approved by the Local Ethics Committee of the Jagiellonian University in Krakow (approval no 1072.6120.180.2021). Written informed consent for participation was not required for this study in accordance with the national legislation and the institutional requirements.

## Author contributions

MO: data collection, imaging review, data analysis, manuscript drafting, manuscript editing and approval, study coordination, KM-S: manuscript editing and approval, AK-K: data collection, statistical analysis, manuscript editing, and approval, IAM: data collection, imaging review, manuscript editing and approval, AS-S: manuscript editing and approval, AH-D: data analysis, manuscript editing and approval.

## Conflict of interest

The authors declare that the research was conducted in the absence of any commercial or financial relationships that could be construed as a potential conflict of interest.

## Publisher’s note

All claims expressed in this article are solely those of the authors and do not necessarily represent those of their affiliated organizations, or those of the publisher, the editors and the reviewers. Any product that may be evaluated in this article, or claim that may be made by its manufacturer, is not guaranteed or endorsed by the publisher.
